# Prognostic impact of CD4-positive T cell subsets in early breast cancer: a study based on the FinHer trial patient population

**DOI:** 10.1186/s13058-018-0942-x

**Published:** 2018-02-26

**Authors:** Marcus Schmidt, Veronika Weyer-Elberich, Jan G. Hengstler, Anne-Sophie Heimes, Katrin Almstedt, Aslihan Gerhold-Ay, Antje Lebrecht, Marco J. Battista, Annette Hasenburg, Ugur Sahin, Konstantine T. Kalogeras, Pirkko-Liisa Kellokumpu-Lehtinen, George Fountzilas, Ralph M. Wirtz, Heikki Joensuu

**Affiliations:** 1 Department of Obstetrics and Gynecology, University Medical Center Mainz, Langenbeckstr. 1, 55131 Mainz, Germany; 2 Institute of Medical Biostatistics, Epidemiology and Informatics (IMBEI), University Medical Center Mainz, Mainz, Germany; 3 Leibniz Research Centre for Working Environment and Human Factors (IfADo) at Dortmund TU, Dortmund, Germany; 4 TRON-Translational Oncology at the University Medical Center Mainz, Mainz, Germany; 5 Laboratory of Molecular Oncology, Hellenic Foundation for Cancer Research/Aristotle University of Thessaloniki, Thessaloniki, Greece; 6 Translational Research Section, Hellenic Cooperative Oncology Group, Athens, Greece; 7 Department of Oncology, Tampere University Hospital and University of Tampere, Tampere, Finland; 8 Aristotle University of Thessaloniki, Thessaloniki, Greece; 9 STRATIFYER Molecular Pathology GmbH, Köln, Germany; 10 Department of Oncology, Helsinki University Hospital and University of Helsinki, Helsinki, Finland

**Keywords:** Breast cancer, Prognosis, Immune system, Humoral, Tumor-infiltrating lymphocytes

## Abstract

**Background:**

The clinical importance of tumor-infiltrating cluster of differentiation 4 (CD4) T cells is incompletely understood in early breast cancer. We investigated the clinical significance of CD4, forkhead box P3 (FOXP3), and B cell attracting chemokine leukocyte chemoattractant-ligand (C-X-C motif) 13 (CXCL13) in early breast cancer.

**Methods:**

The study is based on the patient population of the randomized FinHer trial, where 1010 patients with early breast cancer were randomly allocated to adjuvant chemotherapy containing either docetaxel or vinorelbine, and human epidermal growth factor receptor 2 (HER2)-positive patients were also allocated to trastuzumab or no trastuzumab. Breast cancer CD4, FOXP3, and CXCL13 contents were evaluated using quantitative real-time polymerase chain reaction (qRT-PCR), and their influence on distant disease-free survival (DDFS) was examined using univariable and multivariable Cox regression and Kaplan-Meier estimates in the entire cohort and in selected molecular subgroups. Interactions between variables were analyzed using Cox regression. The triple-negative breast cancer (TNBC) subset of the HE10/97 randomized trial was used for confirmation.

**Results:**

High CXCL13 was associated with favorable DDFS in univariable analysis, and independently in multivariable analysis (HR 0.44, 95% CI 0.29–0.67, *P* ≤ 0.001), most strongly in TNBC (HR 0.39, 95% CI 0.19–0.79, *P* = 0.009). No significant interaction with chemotherapy or trastuzumab administration was detected. Neither tumor CD4 content nor FOXP3 content was associated with DDFS. The favorable prognostic influence of CXCL13 was confirmed in the HE10/97 trial patient population with TNBC (HR 0.30, 95% CI 0.09–0.93; *P* = 0.038).

**Conclusions:**

The results provide a high level of evidence that humoral immunity influences the survival outcomes of patients with early breast cancer, in particular of those with TNBC.

**Trial registration:**

The study reports retrospective biomarker analyses in the prospective FinHer trial and the prospective HE10/97 trial.

ISRCTN76560285. Registered on 18 March 2005.

ACTRN12611000506998. Registered on 16 May 2011.

**Electronic supplementary material:**

The online version of this article (10.1186/s13058-018-0942-x) contains supplementary material, which is available to authorized users.

## Background

Tumor-infiltrating lymphocytes (TILs) likely have an important, albeit still incompletely understood, prognostic and predictive role in breast cancer. For example, a recent meta-analysis of 17 trials with 12,968 patients with breast cancer found that high numbers of TILs were associated with favorable prognosis, and that their numbers were predictive of achieving pathological complete response (pCR) after neoadjuvant chemotherapy [1]. Numerous studies have dealt with the association of different subsets of T cells and prognosis in breast cancer [2]. The importance of cluster of differentiation 8 (CD8)-positive (CD8+) cytotoxic T cells is well-established in breast cancer [3], and CD4+ T cells have a central role in orchestrating antitumoral immunity [4, 5].

The role of different subsets of lymphocytes and their clinical significance in different molecular subgroups of breast cancer is still unclear. To elucidate the prognostic role of different subsets of CD4+ T cells in early breast cancer, we focus here on the CD4+ cells, forkhead box P3 (FOXP3) + CD4+ regulatory T cells (Tregs), and C-X-C motif chemokine ligand 13 (CXCL13)-positive CD4+ follicular helper T (Tfh) cells. FOXP3 drives the development and function of Tregs that are engaged in the maintenance of immunological self-tolerance and in down-regulation of various immune responses [6]. CXCL13, formerly termed B cell-attracting chemokine 1, is a cytokine that belongs to the CXC chemokine family, which selectively attracts C-X-C motif receptor 5-positive B cells (CXCR5) [7]. CXCL13-positive CD4+ Tfh cells are associated with a high frequency of peritumoral tertiary lymphoid structures and generally favorable outcome in breast cancer [8]. Tfh cells expressing CXCL13 were recently found to convert Treg-mediated immune suppression to activation of adaptive antitumor humoral responses in breast cancer [9]. Tfh cells and the CXLC13/CXCR5 axis are crucial for germinal center development and antigen-specific B cell maturation to high-affinity memory cells and antibody-secreting plasma cells [10, 11].

We previously reported an association between a B cell metagene, and, to a lesser extent, a T cell metagene with favorable prognosis in patients with node-negative breast cancer who did not receive adjuvant therapy [12]. The strong favorable impact of the B cell/plasma cell signature on prognosis was later confirmed by others [13]. In a recent comprehensive meta-analysis of expression signatures from ∼ 8000 human tumors, Gentles and co-workers argued that antigen-driven processes required for clonal expansion and emergent humoral immune responses may be important for the prognostic significance of tumor-infiltrating plasma cells [14]. In accordance with this hypothesis, we identified breast tumor-infiltrating plasmablasts and plasma cells using confocal microscopy as the source of immunoglobulin kappa C (IGKC) expression, and found them to be associated with favorable prognosis [15]. However, the role of humoral/B cell-mediated immunity in the development and clinical behavior of breast cancer, or in outcome prediction, is not established, although an increasing body of evidence suggests that humoral immunity, too, is important.

We report here, to our knowledge for the first time, based on a large study that the humoral immune function, as approximated with cancer B cell-attracting chemokine CXCL13 content, is associated with survival in early breast cancer. The findings suggest that B cell-mediated immune functions may be important for the clinical behavior of breast cancer, particularly of the triple-negative subtype.

## Methods

### Study population

In the FinHer trial (identifier ISRCTN76560285) 1010 patients with node-positive or high-risk node-negative breast cancer were randomly assigned to either three cycles of docetaxel or vinorelbine, followed by three cycles of fluorouracil, epirubicin, and cyclophosphamide (FEC) as adjuvant treatments [16]. Patients with human epidermal growth factor receptor 2 (HER2)-positive cancer by chromogenic *in situ* hybridization (CISH) were further randomized to nine weekly trastuzumab infusions, administered concomitantly with chemotherapy, or to no trastuzumab. Patients with hormone receptor-positive cancer received tamoxifen. A review board at the Helsinki University Hospital approved the study. Study participants signed informed consent to allow research assays to be carried out on their tumor tissue. This retrospective biomarker study is reported according to the Reporting Recommendations for Tumor Marker Prognostic Studies (REMARK) criteria [17]. The characteristics of the patients and the tumors are provided in Table 1.

**Table 1:** Associations between median cancer CXCL13 expression and patient and tumor characteristics

Characteristic	PatientsNumber (percentage)	Cancer CXCL13 content		*P*
		≤ MedianNumber (percentage)	> MedianNumber (percentage)	
Tumor size				
pT1	368 (41.8)	172 (46.7%)	196 (44.7%)	
pT2	437 (49.6)	226 (51.7%)	211 (48.3%)	
pT3	76 (8.6)	45 (59.2%)	31 (40.8%)	0.099
Missing data	1			
Axillary nodal status				
pN0	93 (10.6)	45 (48.4%)	48 (51.6%)	
pN1	761 (86.4)	384 (50.5%)	377 (495%)	
pN2	27 (3.1)	14 (51.9%)	13 (48.1%)	0.922
Missing data	1			
Histological grade				
I	127 (15.0)	84 (66.1%)	43 (33.9%)	
II	352 (41.6)	196 (55.7%)	156 (44.3%)	
III	368 (43.4)	147 (39.9%)	221 (60.1%)	< 0.001
Missing data	35			
Age at study entry				
<50 years	393 (44.6)	168 (42.7%)	225 (57.3%)	
≥50 years	489 (55.4)	275 (56.2%)	214 (43.8%)	< 0.001
Estrogen receptor status				
Positive	635 (72.0)	360 (56.7%)	275 (43.3%)	
Negative	247 (28.0)	83 (33.6%)	164 (66.4%)	< 0.001
Progesterone receptor status				
Positive	510 (57.9)	273 (53.5%)	237 (46.5%)	
Negative	371 (42.1)	170 (45.8%)	201 (54.2%)	0.025
Missing data	1			
HER2 status				
Positive	199 (22.6)	80 (40.2%)	119 (59.8%)	
Negative	683 (77.4)	363 (53.1%)	320 (46.9%)	0.002
Ki-67				
≤20% (median)	396 (50.5)	219 (55.3%)	177 (44.7%)	
>20%	388 (49.5)	161 (41.5%)	227 (58.5%)	< 0.001
Missing data	98			
Molecular subtype				
Luminal A-like	331 (40.8)	190 (57.4%)	141 (42.6%)	
Luminal B-like	148 (18.2)	77 (52.0%)	71 (48.0%)	
Triple-negative	134 (16.5)	47 (35.1%)	87 (64.9%)	
HER2-positive	199 24.5)	80 (40.2%)	119 (59.8%)	< 0.001
Missing data	70			
Assigned chemotherapy				
Vinorelbine	437 (49.5)	210 (48.1%)	227 (51.9%)	
Docetaxel	445 (50.5)	233 (52.4%)	212 (47.6%)	0.225
Trastuzumab given (if HER2+) cancers)				
Yes	103 (52.2)	41 (39.8%)	62 (60.2%)	
No	94 (47.7)	38 (40.4%)	56 (59.6%)	0.522

*Abbreviations: CXCL13* C-X-C motif chemokine ligand 13, *HER2* human epidermal growth factor receptor 2

We used triple-negative breast cancer (TNBC) samples from the prospective HE10/97 trial (identifier ACTRN12611000506998) to evaluate the prognostic impact of CXCL13. The Hellenic Cooperative Oncology Group (HeCOG) randomized a total of 595 high-risk patients with breast cancer to postoperative dose-dense sequential chemotherapy with epirubicin, followed by cyclophosphamide, methotrexate, and fluorouracil (CMF) with or without paclitaxel [18]. The clinical protocol and the companion translational research studies were approved by local regulatory authorities and the Bioethics Committee of the Aristotle University of Thessaloniki School of Medicine, respectively. Written informed consent was obtained from all patients. The characteristics of TNBC patients with data available and the tumors are shown in Additional file 1: Table S1.

### Breast cancer biological subtyping

In FinHer, cancer estrogen receptor (ER), progesterone receptor (PR), and human epidermal growth factor receptor-2 (HER2) expression were immunohistochemically assessed according to the institutional guidelines. When HER2 expression was scored 2+ or 3+ (on a scale from 0 to 3+), the number of copies of *HER2* was centrally assessed with CISH [16]. The tumors were considered hormone-receptor-positive when ≥ 10% of cancer cells expressed ER and/or PR. Immunohistochemical assessment for Ki-67 was done locally using a Mib-1 monoclonal antibody (Dako, Glostrup, Denmark). In the HE10/97 trial, tumor ER, PR, HER2, and Ki-67 protein expression was assessed centrally as previously described in detail [18]. Using these markers, breast cancers were stratified as luminal A-like cancers (ER+ and/or PR+, HER2−, Ki-67 ≤ 20%), luminal B-like cancers (ER+ and/or PR+, HER2−, Ki-67 > 20%), HER2-positive cancers (ER and PR positive or negative, HER2+), or triple-negative breast cancers (TNBC; ER−, PR−, and HER2−).

### CD4, FOXP, and CXCL13 assessments

Total RNA was extracted from 5-μm whole formalin-fixed paraffin-embedded (FFPE) tissue sections with ≥ 30% of the section surface area consisting of tumor. Tumor tissue was available in 950 (94.1%) out of the 1010 cases. A sufficient amount of RNA with good quality was isolated from 882 (87.3%) of the FFPE tumor specimens (Additional file 2: Figure S1). CXCL 13, CD4, and FOXP3 was successfully analyzed in 882 (87.3%), 876 (86.7%), and 874 (86.5%) tumors, respectively.

In brief, RNA was first isolated using a fully automated isolation method (XTRACT kit; STRATIFYER Molecular Pathology GmbH, Cologne, Germany) using a liquid handling robot (XTRACT system; STRATIFYER Molecular Pathology GmbH). Following deparaffinization, DNase I digestion steps, and controls for the RNA quality, one-step quantitative real-time (qRT)-PCR was done using a custom-designed gene-specific Taq-Man-based assay to measure cancer CXCL13, CD4, and FOXP3 content. Expression of *CXCL13*, *CD4*, *FOXP3*, and the reference gene *CALM2* mRNA were assessed in triplicates using the SuperScript III Platinum One-Step Quantitative RT-PCR System with ROX (Invitrogen, Karlsruhe, Germany) in a Versant kPCR system (Siemens, Erlangen, Germany). The thermal profile included 30 min at 50 °C, 20.5 min at 8 °C, and 2 min at 95 °C followed by 40 cycles of 15 s at 95 °C, and 30 s at 60 °C. The primer and probe sequences are shown in Additional file 3: Table S2. The relative expression levels of the genes of interest (GOI) were calculated as delta cycle threshold (ΔCt) values (ΔCt = 40 − [CtGOI − Ct (mean of CALM2)]. We stratified the samples using the median breast tumor RNA expression as the cut-off value; 440 cancers had high and 442 had low CXCL13 expression (median, 34.46), 437 had high and 438 had low FOXP3 expression (median, 31.01), and 438 had high and 439 had low CD4 expression (median, 35.30).

To validate the prognostic impact of CXCL13 in an independent cohort, we analyzed TNBC tumor samples from the HE10/97 trial cohort with 595 participants, of whom 366 (62%) had samples available. Of these, 47 (13%) were TNBCs, and sufficient RNA was isolated from 38 (81%) FFPE tumor specimens (Additional file 4: Figure S2). When we stratified the samples using the median breast tumor CXCL13 expression as the cutoff value, 19 TNBCs had high and 19 had low CXCL13 expression (median, 34.21).

### Statistical analysis

The primary objective of this explorative study was to evaluate the associations between CD4-positive T cells and their subsets (CD4+/CXCL13+, CD4+/FOXP3+) with distant disease-free survival (DDFS), which was the survival endpoint in the final analysis of the FinHer trial [16]. The secondary objectives were to study the influence of CD4, CXCL13, and FOXP3 expression in defined molecular subtypes of breast cancer, and the associations with the type of adjuvant therapy administered. DDFS was defined as the time interval between the date of randomization and the date of first cancer recurrence outside of the ipsilateral local region or the date of death, whenever death occurred before distant recurrence. Patients alive without documented evidence of distant metastases were censored at the time of the latest contact. DDFS rates were determined using Kaplan-Meier estimates. Survival was compared between groups using the log-rank test. The potential interactions between tumor CXCL13 expression and the treatment assigned were studied using a Cox proportional hazards model containing the treatment group (docetaxel vs vinorelbine, and trastuzumab vs no trastuzumab when the tumor was HER2-positive), CXCL13 expression (high vs low), and the treatment-by-biomarker interaction term. Frequency tables were analyzed using Fisher’s exact test.

Association between tumor CXCL13, CD4, and FOXP3 expression (tested ≤ median vs > median) and DDFS was investigated using univariable and multivariable Cox proportional hazards models. Other covariables in the multivariable models were age at the time of study entry (≤50 vs >50 years), breast tumor size (pT1 vs pT2 vs pT3/T4), axillary nodal status (pN0 vs pN1 vs pN2), histological grade of differentiation (grade I vs II vs III), HER2 status (positive vs negative), ER status (positive vs negative), PR status (positive vs negative), tumor Ki-67 expression (≤20% vs >20%), and cancer molecular subtype (luminal A-like vs luminal B-like vs TNBC vs HER2-positive).

All *P* values are two-tailed, and *P* < 0.05 was considered significant. As all analyses are explorative and not adjusted for multiple testing, the *P* values should be interpreted with caution and in connection with the effect estimates. Statistical analyses were performed using the Statistical Package for Social Science (SPSS) (SPSS Inc., version 22, Chicago, IL, USA).

## Results

### Cancer biological subtypes

Of the 883 cancers, 331 (37.5%) were luminal A-like, 148 (16.8%) luminal B-like, 199 (22.5%) HER2-positive, 134 (15.2%) TNBC, and in 71 (8.0%) cases the subtype could not be determined due to missing data on cancer Ki-67 expression.

### Associations with patient and cancer characteristics

Cancer CD4, CXCL13, and FOXP3 expression correlated positively with each other (*P* ≤ 0.001). Breast tumor expression of CXCL13 (Table 1) and of FOXP3 (Additional file 5: Table S3) above the median value were both associated with poor histological grade of differentiation, negative ER and PR status, positive HER2 status, Ki-67 expression above the median value, and with the HER2-positive and the TNBC molecular subtypes, whereas CD4 (Additional file 6: Table S4) expression was not associated with these variables.

### Survival analyses

**Fig. 1:**
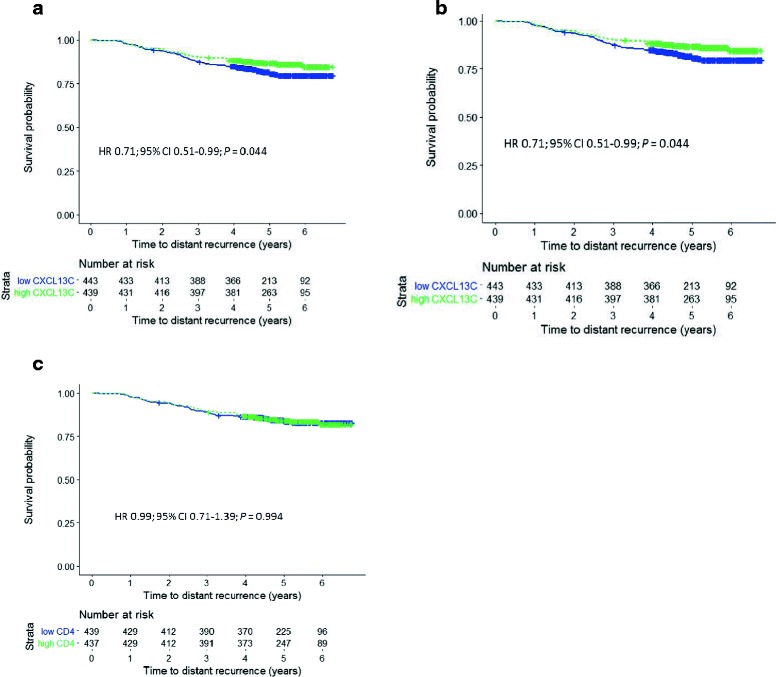
Influence of the median breast cancer CXCL13 content (**a**), forkhead box P3 (FOXP3) content (**b**), and CD4 content (**c**) on distant disease-free survival in the FinHER trial

Cancer CXCL13 expression above the median value was associated with favorable DDFS (HR 0.71, 95% CI 0.51–0.99; *P* = 0.044), high FOXP3 content tended to be associated with unfavorable DDFS (HR 1.33, 95% CI 0.95–1.86; *P* = 0.094), and tumor CD4 content was not associated with DDFS (HR 0.99, 95% CI 0.71–1.39; *P* = 0.994; Fig. 1). As expected, the standard prognostic factors were also associated with DDFS (Additional file 7: Table S5). When the potential interactions between cancer CXCL13 content and systemic treatments with DDFS were examined, there was no significant interaction with the type of chemotherapy given or with administration versus no administration of trastuzumab (*P*_interaction_ = 0.255 and 0.325, respectively).

In a multivariable analysis high breast tumor median CXCL13 content was independently associated with favorable DDFS (HR 0.44, 95% CI 0.29–0.67; *P* ≤ 0.001), whereas CD4 content and FOXP3 content were not (HR 1.89, 95% CI 0.80–1.78; *P* = 0.396; and HR 1.20, 95% CI 0.78–1.84; *P* = 0.400, respectively). Besides CXCL13, only the axillary nodal status and the molecular subtypes had independent prognostic value (Table 2).

**Table 2:** Multivariable Cox regression analysis for distant disease-free survival

Covariable	Distant disease-free survivalHR (95% CI)	*P*
CXCL13 expression		
≤ Median	ref.	
> Median	0.44 (0.29–0.67)	< 0.001
FOXP3 expression		
≤ Median	ref.	0.400
> Median	1.20 (0.78–1.84)	
CD4 expression		
≤ Median	ref.	0.396
> Median	1.89 (0.80–1.78)	
Age at study entry		
≤50 years	Ref.	0.516
>50 years	0.88 (0.62–1.27)	
Tumor size		0.105
pT1	Ref.	
pT2	1.22 (0.82–1.81)	0.330
pT3	1.89 (1.05–3.40)	0.034
Axillary nodal status		< 0.001
pN0	Ref.	
pN1	4.51 (1.94–10.52)	< 0.001
pN2	14.27 (4.78–42.71)	< 0.001
Histological grade		0.229
I	Ref.	
II	2.06 (0.86–4.95)	0.105
III	2.22 (0.88–5.52)	0.088
Molecular subtype		< 0.001
Luminal A-like	Ref.	
Luminal B-like	1.79 (1.01–3.14)	0.045
Triple-negative	4.18 (2.30–7.59)	< 0.001
HER2-positive	2.71 (1.56–4.68)	< 0.001

*Abbreviations: CD4* cluster of differentiation 4, *CI* confidence interval, *CXCL13* C-X-C motif chemokine ligand 13, *FOXP3* forkhead box P3, *HER2* human epidermal growth factor receptor 2, *HR* hazard ratio

### Influence on survival in molecular subsets

**Fig. 2:**
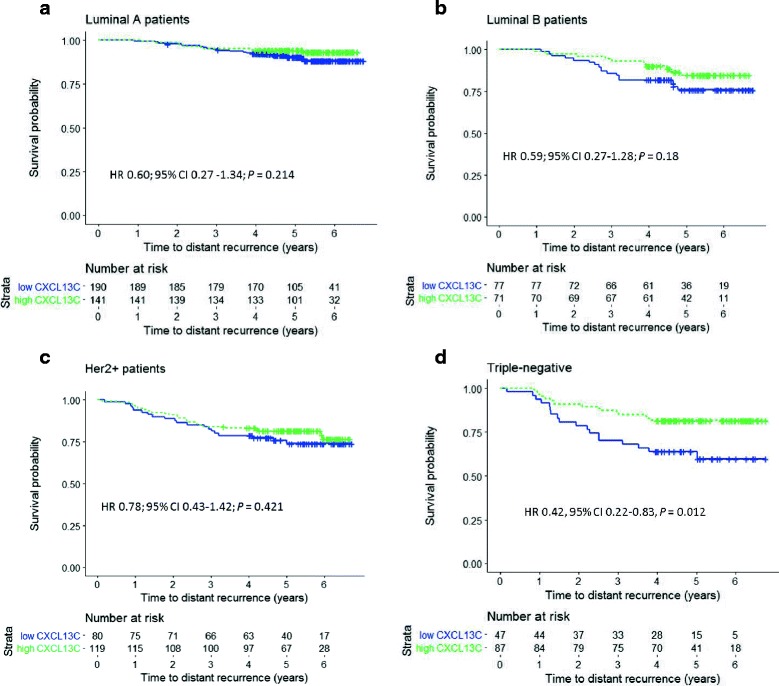
Influence of the median breast cancer CXCL13 content on distant disease-free survival in luminal A-like cancer (**a**), luminal B-like cancer (**b**), human epidermal growth factor receptor 2 (HER2)-positive cancer (**c**), and triple-negative cancer (**d**) in the FinHER trial

The influence of cancer CXCL13 content on DDFS was most evident in the TNBC subset (Table 3). In univariable survival analyses, high tumor CXCL13 content was significantly associated with favorable DDFS in TNBC (HR 0.42, 95% CI 0.22–0.83, *P* = 0.012) unlike the other molecular subtypes (Fig. 2). Tumor CXCL13 content also had an independent influence on DDFS in a multivariable analysis adjusted for age at the time of study entry, tumor size, axillary nodal status, and histological grade of differentiation (HR 0.39, 95% CI 0.19–0.79; *P* = 0.009; Table 3).

**Table 3:** Association between breast cancer median CXCL13 content and distant disease-fee survival in four molecular subtypes

Subtype	Univariable modelHR (95% CI)	*P*	Multivariable modelHR (95% CI)	*P*
Luminal A-like	0.60 (0.27–1.34)	0.214	0.57 (0.24–1.34)	0.194
Luminal B-like	0.59 (0.27–1.28)	0.180	0.49 (0.20–1.17)	0.109
Triple-negative	0.42 (0.22–0.83)	0.012	0.39 (0.19–0.79)	0.009
HER2-positive	0.78 (0.43–1.42)	0.421	0.75 (0.40–1.41)	0.372

*Abbreviations: CI* confidence interval, *HR* hazard ratio, *HER2* human epidermal growth factor receptor 2

Unlike CXCL13, FOXP3 (Additional file 8: Table S6) and CD4 (Additional file 9: Table S7) content in tumor tissue was not significantly associated with DDFS in any of the four molecular subtypes, respectively.

**Fig. 3:**
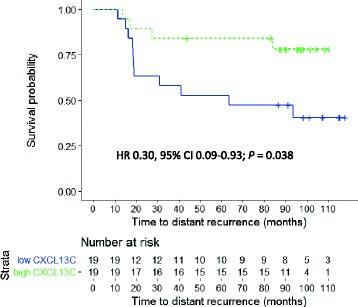
Influence of the median breast cancer CXCL13 content on distant disease-free survival in triple-negative cancer in the HE1097 trial

The association between high median tumor CXCL13 content and favorable DDFS in TNBC was confirmed in the HE10/97 trial population (HR 0.30, 95% CI 0.09–0.93; *P* = 0.038) (Fig. 3).

## Discussion

High tumor CXCL13 expression was associated with several factors that are generally associated with poor survival in breast cancer, such as low cancer histological grade of differentiation, high Ki-67 expression, and negative ER expression in the FinHer trial patient population. Despite this, high cancer CXCL13 expression was associated with favorable DDFS in univariable survival analysis, and tumor CXCL13 expression had an independent influence on survival also in a multivariable model. The prognostic influence was most marked in the subset of patients with TNBC, which we confirmed in an independent series of patients with early breast cancer enrolled in the HE10/97 trial.

CXCL13+/CD4+ Tfh cells are crucial for germinal center development, and their presence is associated with extensive lymphocyte infiltration and formation of peritumoral tertiary lymphoid follicles [8]. In a study on early breast cancer in which the patients had not received systemic treatment, presence of CXCL13 Tfh cells in cancer predicted longer survival and a high pCR rate after preoperative chemotherapy [8]. Similarly, in the neoadjuvant GeparSixto trial a high cancer CXCL13 mRNA content was associated with a high pCR rate [19]. We and others have found that in retrospective studies high expression of B cell-related transcripts is associated with favorable survival in early breast cancer [12–15, 20–22]. To our knowledge this study is the first one showing that in a large randomized trial patient population high B cell-attracting chemokine CXCL13 is independently associated with favorable survival outcomes, indicating an important role of the humoral immune response in disease control among patients with early breast cancer.

We [23] and others [8] have reported significant association between high tumor CXCL13 content and favorable survival among patients with HER2-positive cancer not treated with trastuzumab. We did not confirm this finding in the present study in which half of the patients were treated with trastuzumab. The host immune system likely contributes to trastuzumab efficacy [24]. Loi and co-workers did not identify a significant association between the numbers of TILs and DDFS among patients with HER2-positive cancer in the FinHer patient population, but detected an interaction between a high tumor TIL content and trastuzumab benefit [25]. The association between TILs and the benefit from trastuzumab is controversial, as conflicting results were obtained from the N9831 trial [26]. In the present analysis, we did not find a significant interaction between cancer CXCL13 expression and trastuzumab treatment. Potential explanations for these seemingly conflicting results in HER2-positive disease include differences in the study methodology (TILs were assessed either immunohistochemically or by gene expression analyses), the components of the immune system addressed differed (CXCL13 assays reflect mainly the humoral immunity only), and differences in the trial designs that may have influenced the immune function (in FinHer trastuzumab was administered up front, in N9831 doxorubicin plus cyclophosphamide were administered prior to starting trastuzumab).

The current results in TNBC are well in an agreement with previous findings. A high tumor TIL content has been found to be associated with favorable survival in TNBC [25, 27, 28]. Using DNA microarray gene expression analysis we demonstrated that three “coordinates in breast cancer” - proliferation, ER, and the immune system - facilitate orientation and help to correctly interpret breast cancer biology. Based on the extent of immune-related transcripts we defined two subtypes of basal-like breast cancer, namely basal-like A (advantageous prognosis) and basal-like B (bad prognosis) [29]. Later, this notion of the importance of immune infiltrates especially in triple-negative breast cancer was convincingly refined and elaborated by Lehmann and co-workers [30, 31]. The association between high cancer TIL content and survival in TNBC may not be surprising, since the overall mutation rate is the highest in the basal-like and HER2-enriched subtypes [32]. A high mutation load leads to an increased presentation of neo-antigens to immune cells resulting in pronounced lymphocytic infiltrates, which may be a biomarker for cancer immunotherapy [33]. A recent retrospective analysis, however, challenged this association and reported that lymphocyte-rich TNBC had significantly lower mutation and neo-antigen counts than lymphocyte-poor TNBC [34]. Nonetheless, most of the immunogenic mutanome is recognized by the CD4+ T cells, and vaccination with CD4+ immunogenic mutations leads to strong antitumor activity [35, 36]. These findings may have important potential implications for breast cancer immunotherapy. The first results with immune checkpoint inhibitors, such as pembrolizumab that target the programmed cell death protein 1 (PD-1), seem encouraging with clinical responses in some heavily pretreated patients with advanced TNBC [37]. Evaluation of the B cell response biomarker CXCL13 in addition to PD-L1 testing might be useful to further enrich such responsive patients.

Unlike CXCL13, the median tumor CD4 or FOXP3 contents were not significantly associated with DDFS. Since CD4 is expressed in many T cell subsets including T helper 1 (Th1) cells, T helper 2 (Th2) cells, T helper 17 (Th17) cells, Tfh cells, and Tregs, each of which may have a different impact on prognosis, this observation may be expected. Prior studies based on immunohistochemical assessment that have addressed the prognostic significance of FOXP3+ Tregs have shown conflicting results [38–41].

The potential weakness of the study is that it was exploratory, and that we may have lacked the statistical power to detect small effects on survival, in particular in the molecular subgroups. The systemic therapy consisted of several agents, which is a potential confounding factor, as the agents might have varying effects on the immune system. The strength of the study is that we report the prognostic significance of CD4+ T cell subsets in a large randomized trial, and confirmed the prognostic impact of CXCL13 in TNBC in an independent cohort. This allows a high level of evidence when evaluating prognostic or predictive biomarkers [42].

## Conclusions

The present results suggest that the humoral immune function has prognostic significance in early breast cancer. The prognostic effect of cancer CXCL13 content was most pronounced in the subset of patients with TNBC. In general, the current results provide a high level of evidence for the independent prognostic role of CXCL13 and lend support for immunotherapeutic interventions that may strengthen the immune response, especially in patients with TNBC.

## Additional files

Additional file 1: **Table S1.** Associations between median cancer CXCL13 expression and patient and tumor characteristics in triple-negative patients enrolled in HE10/97. (DOCX 15 kb)

Additional file 2: **Figure S1.** A CONSORT diagram showing patient selection for the study (FinHER). (PPT 106 kb)

Additional file 3: **Table S2.** The primer and probe sequences used for mRNA quantification. (DOCX 13 kb)

Additional file 4: **Figure S2.** A CONSORT diagram showing patient selection for the study (HE10/97). (PPT 103 kb)

Additional file 5: **Table S3.** Associations between patient and tumor characteristics and cancer median FOXP3 expression. (DOCX 17 kb)

Additional file 6: **Table S4.** Associations between patient and tumor characteristics and cancer median CD4 expression. (DOCX 16 kb)

Additional file 7: **Table S5.** Univariable Cox regression analysis for distant disease-free survival. (DOCX 16 kb)

Additional file 8: **Table S6.** Associations between breast cancer median FOXP3 content and cancer molecular subtype in univariable and multivariable cox regression models for distant disease-free survival. (DOCX 14 kb)

Additional file 9: **Table S7.** Associations between breast cancer median CD4 content and cancer molecular subtype in univariable and multivariable cox regression models for distant disease-free survival. (DOCX 14 kb)

## Abbreviations

CD4: Cluster of differentiation 4
CI: Confidence interval
CISH: Chromogenic *in situ* hybridization
CXCL13: C-X-C motif chemokine ligand 13
DDFS: Distant disease-free survival
ER: Estrogen receptor
FFPE: Formalin-fixed paraffin-embedded
FOXP3: Forkhead box P3
HER2: Human epidermal growth factor receptor 2
HR: Hazard ratio
PR: Progesterone receptor
qRT-PCR: Quantitative real-time PCR
Tfh: Follicular helper T cells
TILs: Tumor-infiltrating lymphocytes
TNBC: Triple-negative breast cancer
Tregs: Regulatory T cells

## References

[1] Yu X, Zhang Z, Wang Z, Wu P, Qiu F, Huang J (2016). Prognostic and predictive value of tumor-infiltrating lymphocytes in breast cancer: a systematic review and meta-analysis. Clin Transl Oncol..

[2] Burugu S, Asleh-Aburaya K, Nielsen TO (2017). Immune infiltrates in the breast cancer microenvironment: detection, characterization and clinical implication. Breast Cancer..

[3] Mahmoud SMA, Paish EC, Powe DG, Macmillan RD, Grainge MJ, Lee AHS (2011). Tumor-infiltrating CD8+ lymphocytes predict clinical outcome in breast cancer. J Clin Oncol..

[4] Knutson KL, Disis ML (2005). Tumor antigen-specific T helper cells in cancer immunity and immunotherapy. Cancer Immunol Immunother..

[5] Schoenberger SP, Toes RE, van der Voort EI, Offringa R, Melief CJ (1998). T-cell help for cytotoxic T lymphocytes is mediated by CD40-CD40L interactions. Nature..

[6] Yagi H, Nomura T, Nakamura K, Yamazaki S, Kitawaki T, Hori S (2004). Crucial role of FOXP3 in the development and function of human CD25+CD4+ regulatory T cells. Int Immunol..

[7] Legler DF, Loetscher M, Roos RS, Clark-Lewis I, Baggiolini M, Moser B (1998). B cell-attracting chemokine 1, a human CXC chemokine expressed in lymphoid tissues, selectively attracts B lymphocytes via BLR1/CXCR5. J Exp Med..

[8] Gu-Trantien C, Loi S, Garaud S, Equeter C, Libin M, de Wind A (2013). CD4(+) follicular helper T cell infiltration predicts breast cancer survival. J Clin Invest..

[9] Gu-Trantien C, Migliori E, Buisseret L, de Wind A, Brohee S, Garaud S, et al. CXCL13-producing TFH cells link immune suppression and adaptive memory in human breast cancer. JCI Insight. 2017;2 10.1172/jci.insight.91487 PMC5453706 28570278

[10] Ansel KM, Ngo VN, Hyman PL, Luther SA, Forster R, Sedgwick JD (2000). A chemokine-driven positive feedback loop organizes lymphoid follicles. Nature..

[11] Nutt SL, Tarlinton DM (2011). Germinal center B and follicular helper T cells: siblings, cousins or just good friends?. Nat Immunol..

[12] Schmidt M, Bohm D, von Torne C, Steiner E, Puhl A, Pilch H (2008). The humoral immune system has a key prognostic impact in node-negative breast cancer. Cancer Res..

[13] Bianchini G, Qi Y, Alvarez RH, Iwamoto T, Coutant C, Ibrahim NK (2010). Molecular anatomy of breast cancer stroma and its prognostic value in estrogen receptor-positive and -negative cancers. J Clin Oncol..

[14] Gentles AJ, Newman AM, Liu CL, Bratman SV, Feng W, Kim D (2015). The prognostic landscape of genes and infiltrating immune cells across human cancers. Nat Med..

[15] Schmidt M, Hellwig B, Hammad S, Othman A, Lohr M, Chen Z (2012). A comprehensive analysis of human gene expression profiles identifies stromal immunoglobulin kappa C as a compatible prognostic marker in human solid tumors. Clin Cancer Res..

[16] Joensuu H, Bono P, Kataja V, Alanko T, Kokko R, Asola R (2009). Fluorouracil, epirubicin, and cyclophosphamide with either docetaxel or vinorelbine, with or without trastuzumab, as adjuvant treatments of breast cancer: final results of the FinHer Trial. J Clin Oncol..

[17] McShane LM, Altman DG, Sauerbrei W, Taube SE, Gion M, Clark GM (2005). Reporting recommendations for tumor marker prognostic studies. J Clin Oncol..

[18] Fountzilas G, Skarlos D, Dafni U, Gogas H, Briasoulis E, Pectasides D (2005). Postoperative dose-dense sequential chemotherapy with epirubicin, followed by CMF with or without paclitaxel, in patients with high-risk operable breast cancer: a randomized phase III study conducted by the Hellenic Cooperative Oncology Group. Ann Oncol..

[19] Denkert C, von Minckwitz G, Brase JC, Sinn BV, Gade S, Kronenwett R (2015). Tumor-infiltrating lymphocytes and response to neoadjuvant chemotherapy with or without carboplatin in human epidermal growth factor receptor 2-positive and triple-negative primary breast cancers. J Clin Oncol..

[20] Chen Z, Gerhold-Ay A, Gebhard S, Boehm D, Solbach C, Lebrecht A (2012). Immunoglobulin kappa C predicts overall survival in node-negative breast cancer. PLoS One..

[21] Rody A, Karn T, Liedtke C, Pusztai L, Ruckhaeberle E, Hanker L (2011). A clinically relevant gene signature in triple negative and basal-like breast cancer. Breast Cancer Res..

[22] Heimes A, Madjar K, Edlund K, Battista MJ, Almstedt K, Gebhard S (2017). Prognostic significance of interferon regulating factor 4 (IRF4) in node-negative breast cancer. J Cancer Res Clin Oncol..

[23] Heimes A, Madjar K, Edlund K, Battista MJ, Almstedt K, Elger T (2017). Subtype-specific prognostic impact of different immune signatures in node-negative breast cancer. Breast Cancer Res Treat..

[24] Bianchini G, Gianni L (2014). The immune system and response to HER2-targeted treatment in breast cancer. Lancet Oncol..

[25] Loi S, Michiels S, Salgado R, Sirtaine N, Jose V, Fumagalli D (2014). Tumor infiltrating lymphocytes are prognostic in triple negative breast cancer and predictive for trastuzumab benefit in early breast cancer: results from the FinHER trial. Ann Oncol..

[26] Perez EA, Ballman KV, Tenner KS, Thompson EA, Badve SS, Bailey H (2016). Association of stromal tumor-infiltrating lymphocytes with recurrence-free survival in the N9831 adjuvant trial in patients with early-stage HER2-positive breast cancer. JAMA Oncol..

[27] Loi S, Sirtaine N, Piette F, Salgado R, Viale G, van Eenoo F (2013). Prognostic and predictive value of tumor-infiltrating lymphocytes in a phase III randomized adjuvant breast cancer trial in node-positive breast cancer comparing the addition of docetaxel to doxorubicin with doxorubicin-based chemotherapy: BIG 02-98. J Clin Oncol..

[28] Adams S, Gray RJ, Demaria S, Goldstein L, Perez EA, Shulman LN (2014). Prognostic value of tumor-infiltrating lymphocytes in triple-negative breast cancers from two phase III randomized adjuvant breast cancer trials: ECOG 2197 and ECOG 1199. J Clin Oncol..

[29] Schmidt M, Hengstler JG, von Torne C, Koelbl H, Gehrmann MC (2009). Coordinates in the universe of node-negative breast cancer revisited. Cancer Res..

[30] Lehmann BD, Bauer JA, Chen X, Sanders ME, Chakravarthy AB, Shyr Y (2011). Identification of human triple-negative breast cancer subtypes and preclinical models for selection of targeted therapies. J Clin Invest..

[31] Lehmann BD, Jovanovic B, Chen X, Estrada MV, Johnson KN, Shyr Y (2016). Refinement of triple-negative breast cancer molecular subtypes: implications for neoadjuvant chemotherapy selection. PLoS One..

[32] Cancer Genome Atlas Network. Comprehensive molecular portraits of human breast tumours. Nature. 2012;490:61–70. 10.1038/nature11412 PMC3465532 23000897

[33] Schumacher TN, Schreiber RD (2015). Neoantigens in cancer immunotherapy. Science..

[34] Karn T, Jiang T, Hatzis C, Sanger N, El-Balat A, Rody A (2017). Association between genomic metrics and immune infiltration in triple-negative breast cancer. JAMA Oncol..

[35] Kreiter S, Vormehr M, van de Roemer N, Diken M, Lower M, Diekmann J (2015). Mutant MHC class II epitopes drive therapeutic immune responses to cancer. Nature..

[36] Sahin U, Derhovanessian E, Miller M, Kloke B, Simon P, Lower M (2017). Personalized RNA mutanome vaccines mobilize poly-specific therapeutic immunity against cancer. Nature..

[37] Nanda R, Chow LQM, Dees EC, Berger R, Gupta S, Geva R (2016). Pembrolizumab in patients with advanced triple-negative breast cancer: phase Ib KEYNOTE-012 study. J Clin Oncol..

[38] Bates GJ, Fox SB, Han C, Leek RD, Garcia JF, Harris AL (2006). Quantification of regulatory T cells enables the identification of high-risk breast cancer patients and those at risk of late relapse. J Clin Oncol..

[39] Liu S, Foulkes WD, Leung S, Gao D, Lau S, Kos Z (2014). Prognostic significance of FOXP3+ tumor-infiltrating lymphocytes in breast cancer depends on estrogen receptor and human epidermal growth factor receptor-2 expression status and concurrent cytotoxic T-cell infiltration. Breast Cancer Res..

[40] Mahmoud SMA, Paish EC, Powe DG, Macmillan RD, Lee AHS, Ellis IO (2011). An evaluation of the clinical significance of FOXP3+ infiltrating cells in human breast cancer. Breast Cancer Res Treat..

[41] West NR, Kost SE, Martin SD, Milne K, Deleeuw RJ, Nelson BH (2013). Tumour-infiltrating FOXP3(+) lymphocytes are associated with cytotoxic immune responses and good clinical outcome in oestrogen receptor-negative breast cancer. Br J Cancer..

[42] Simon RM, Paik S, Hayes DF (2009). Use of archived specimens in evaluation of prognostic and predictive biomarkers. J Natl Cancer Inst..

