# Supplementary material for: Prognostic impact of CD4-positive T cell subsets in early breast cancer: a study based on the FinHer trial patient population
Source: Breast Cancer Res. 2018 Feb 26;20:15. doi: 10.1186/s13058-018-0942-x (PMC5827982; doi:10.1186/s13058-018-0942-x)
Supplement: Supplementary file 1 — Table S1. Associations between median cancer CXCL13 expression and patient and tumor characteristics in triple-negative patients enrolled in HE10/97. (DOCX 15 kb) [file 13058_2018_942_MOESM1_ESM.docx]

**Table S1**: Associations Between Median Cancer CXCL13 Expression And Patient And Tumor Characteristics In Triple-Negative Patients Enrolled In HE10/97

| **Characteristic** | **Patients**  **n (%)**  **n=47** | **CXCL13**  **Low n (%) High n (%)**  **n=19 n=19** | | **P** |
| --- | --- | --- | --- | --- |
| **Age at study entry** |  |  |  |  |
| <50 years | 21 (44.7) | 9 (47.4) | 9 (47.4) |  |
| ≥50 years | 26 (55.3) | 10 (52.6) | 10 (52.6) | 1 |
| **Tumor Size** |  |  |  |  |
| T1 | 16 (34.0) | 6 (31.6) | 6 (31.6) |  |
| T2 | 19 (40.4) | 8 (42.1) | 9 (47.4) |  |
| T3 | 12 (25.6) | 5 (26.3) | 4 (21.1) | 1 |
| **Axillary nodal status** |  |  |  |  |
| N0 | 2 (4.3) | 0 (0) | 1 (5.3) |  |
| N1 | 15 (31.9) | 5 (26.3) | 8 (42.1) |  |
| N2 | 12 (25.5) | 4 (21.1) | 6 (31.6) |  |
| N3 | 18 (38.3) | 10 (52.6) | 4 (21.1) | 0.171 |
| **Histological grade** |  |  |  |  |
| Grade I | 1 (2.1) | 1 (5.2) | 0 (0) |  |
| Grade II | 15 (31.9) | 9 (47.4) | 3 (15.8) |  |
| Grade III | 31 (66.0) | 9 (47.4) | 16 (84.2) | 0.038 |
| **Study group** |  |  |  |  |
| E-CMF | 24 (63.2) | 14 (73.7) | 10 (52.6) |  |
| E-T-CMF | 14 (36.8) | 5 (26.3) | 9 (47.4) | 0.313 |

Abbreviations: CMF; cyclophosphamide / methotrexate / 5-fluorouracil; CXCL13, C-X-C motif chemokine ligand 13; E, epirubicin; T, paclitaxel.
