# Supplementary material for: Prognostic impact of CD4-positive T cell subsets in early breast cancer: a study based on the FinHer trial patient population
Source: Breast Cancer Res. 2018 Feb 26;20:15. doi: 10.1186/s13058-018-0942-x (PMC5827982; doi:10.1186/s13058-018-0942-x)
Supplement: Supplementary file 2 — Figure S1. A CONSORT diagram showing patient selection for the study (FinHER). (PPT 106 kb) [file 13058_2018_942_MOESM2_ESM.ppt]

## Slide 1
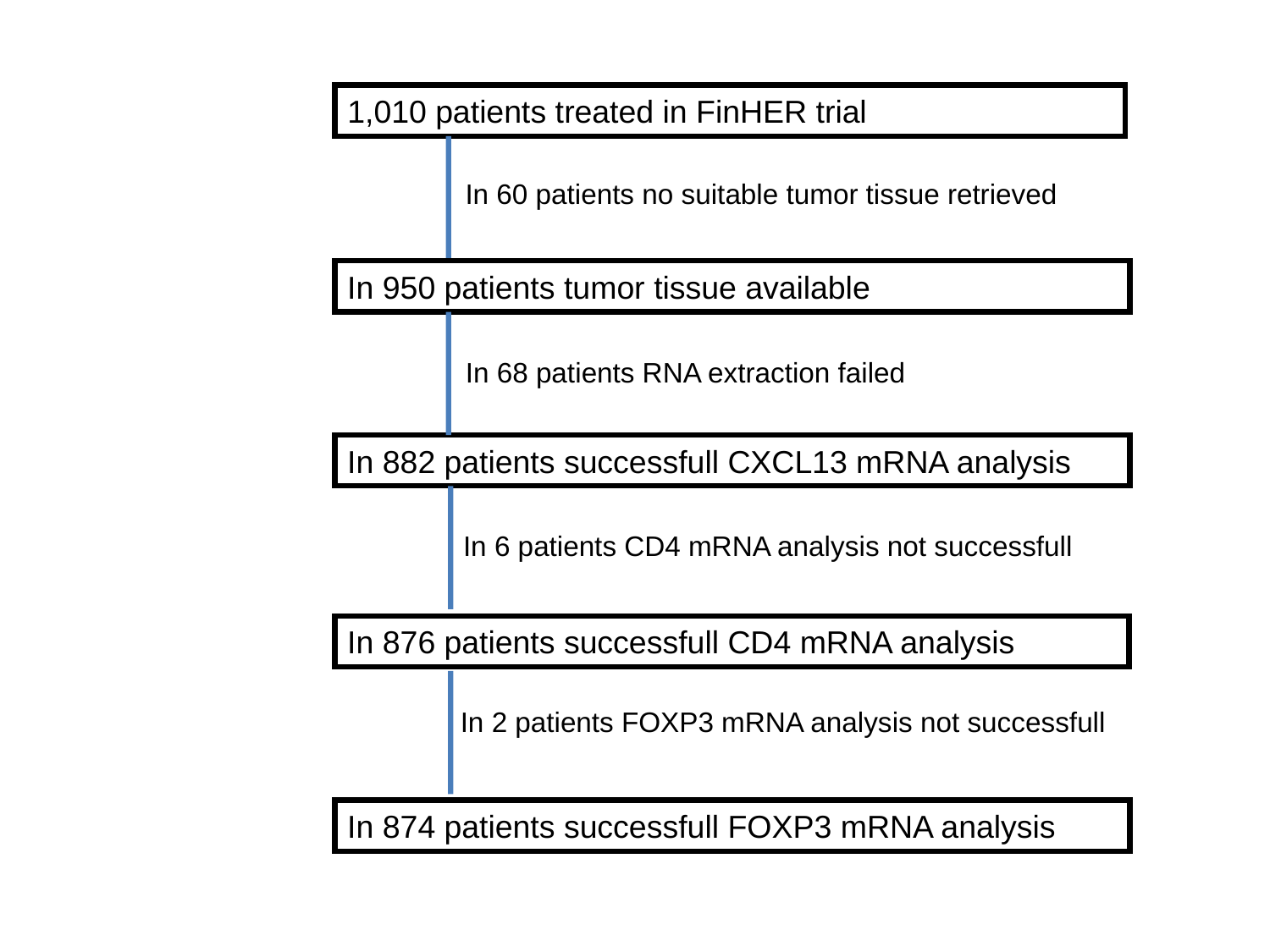

1,010 patients treated in FinHER trial
In 60 patients no suitable tumor tissue retrieved
In 950 patients tumor tissue available
In 68 patients RNA extraction failed
In 882 patients successfull CXCL13 mRNA analysis
In 6 patients CD4 mRNA analysis not successfull
In 876 patients successfull CD4 mRNA analysis
In 2 patients FOXP3 mRNA analysis not successfull
In 874 patients successfull FOXP3 mRNA analysis
