# Supplementary material for: Prognostic impact of CD4-positive T cell subsets in early breast cancer: a study based on the FinHer trial patient population
Source: Breast Cancer Res. 2018 Feb 26;20:15. doi: 10.1186/s13058-018-0942-x (PMC5827982; doi:10.1186/s13058-018-0942-x)
Supplement: Supplementary file 3 — Table S2. The primer and probe sequences used for mRNA quantification. (DOCX 13 kb) [file 13058_2018_942_MOESM3_ESM.docx]

**Table S2.** The Primer And Probe Sequences Used for mRNA Quantification

| **Target gene** | **Probe** | **Forward Primer** | **Reverse Primer** |
| --- | --- | --- | --- |
| *CD4* | ACATCAAGGTTCTGCCCACATGGTCCACCC | GTGGCAGTGTCTGCTGAGTGA | AGCACAATCAGGGCCATTG |
| *CXCL13* | TGGTCAGCAGCCTCTCTCCAGTCCA | CGACATCTCTGCTTCTCATGCT | AGCTTGTGTAATAGACCTCCAGAACA |
| *FOXP3* | TTTTCTGTCAGTCCACTTCACCAAGCCTG | CCCACAAGCCAGGCTGAT | GCATCGGGTCCTTGTCCA |

Abbreviations: CD4, cluster of differentiation 4; CXCL13, C-X-C motif chemokine ligand 13; FOXP3, forkhead box P3.
