# Supplementary material for: Prognostic impact of CD4-positive T cell subsets in early breast cancer: a study based on the FinHer trial patient population
Source: Breast Cancer Res. 2018 Feb 26;20:15. doi: 10.1186/s13058-018-0942-x (PMC5827982; doi:10.1186/s13058-018-0942-x)
Supplement: Supplementary file 4 — Figure S2. A CONSORT diagram showing patient selection for the study (HE10/97). (PPT 103 kb) [file 13058_2018_942_MOESM4_ESM.ppt]

## Slide 1
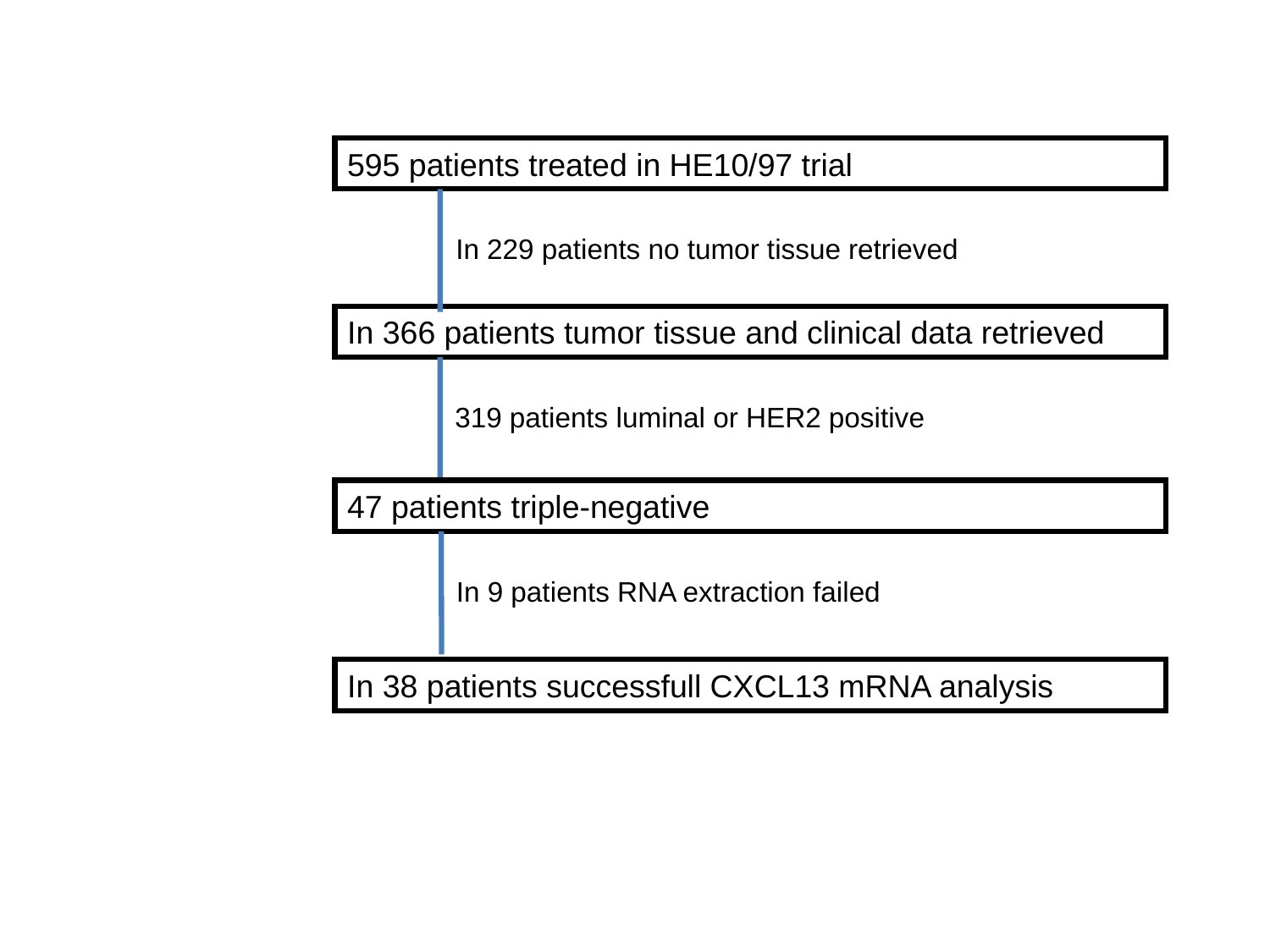

595 patients treated in HE10/97 trial
In 229 patients no tumor tissue retrieved
In 366 patients tumor tissue and clinical data retrieved
319 patients luminal or HER2 positive
47 patients triple-negative
In 9 patients RNA extraction failed
In 38 patients successfull CXCL13 mRNA analysis
