# Supplementary material for: Prognostic impact of CD4-positive T cell subsets in early breast cancer: a study based on the FinHer trial patient population
Source: Breast Cancer Res. 2018 Feb 26;20:15. doi: 10.1186/s13058-018-0942-x (PMC5827982; doi:10.1186/s13058-018-0942-x)
Supplement: Supplementary file 5 — Table S3. Associations between patient and tumor characteristics and cancer median FOXP3 expression. (DOCX 17 kb) [file 13058_2018_942_MOESM5_ESM.docx]

**Table S3**. Associations Between Patient And Tumor Characteristics And Cancer Median FOXP3 Expression

| **Characteristic** | **Cancer FOXP3 Content**  **≤ Median > Median No. (%) No. (%)** | | ***P*** |
| --- | --- | --- | --- |
|  |  |  |  |
| Tumor size |  |  | 0.043 |
| pT1 | 191 (52.0%) | 175 (47.8%) |  |
| pT2 | 200 (46.4%) | 231 (53.6%) |  |
| pT3 | 46 (60.5%) | 30 (39.5%) |  |
| Axillary nodal status |  |  | 0.027 |
| pN0 | 34 (37.0%) | 58 (63.0%) |  |
| pN1 | 389 (51.5%) | 366 (48.5%) |  |
| pN2 | 14 (53.8%) | 12 (46.2%) |  |
| Histological grade |  |  | ≤0.001 |
| I | 93 (73.2%) | 34 (26.8%) |  |
| II | 199 (57.5%) | 147 (42.5%) |  |
| III | 123 (33.5%) | 244 (66.5%) |  |
| Age at study entry |  |  | 0.946 |
| <50 years | 197 (50.3%) | 195 (49.7%) |  |
| ≥50 years | 240 (49.8%) | 242 (50.2%) |  |
| Estrogen receptor status |  |  | ≤0.001 |
| Positive | 364 (57.8%) | 266 (42.2%) |  |
| Negative | 73 (29.9%) | 171 (70.1%) |  |
| Progesterone receptor status |  |  | ≤0.001 |
| Positive | 300 (59.3%) | 206 (40.7%) |  |
| Negative | 137 (37.3%) | 230 (62.7%) |  |
| HER2 status |  |  | ≤0.001 |
| Positive | 51 (25.9%) | 146 (74.1%) |  |
| Negative | 386 (57.0%) | 291 (43.0%) |  |
| Ki-67 |  |  | ≤0.001 |
| ≤ 20% (median) | 244 (62.2%) | 148 (37.8%) |  |
| > 20% | 124 (32.3%) | 260 (67.7%) |  |
| Molecular subtype  L |  |  | ≤0.001 |
| Luminal A-like | 220 (67.1%) | 108 (32.9%) |  |
| Luminal B-like | 68 (46.3%) | 79 (53.7%) |  |
| Triple-negative | 46 (34.8%) | 86 (65.2%) |  |
| HER2-positive | 51 (25.9%) | 146 (74.1%) |  |
| Assigned chemotherapy |  |  | 0.543 |
| Vinorelbine | 211 (48.8%) | 221 (51.2%) |  |
| Docetaxel | 226 (51.1%) | 216 (48.9%) |  |
| Trastuzumab given (if HER2+) cancers) |  |  | 0.417 |
| Yes | 24 (23.5%) | 78 (76.5%) |  |
| No | 27 (29.0%) | 66 (71.0%) |  |

Abbreviations: FOXP3, forkhead box P3; HER2, human epidermal growth factor receptor 2.
