# Supplementary material for: Prognostic impact of CD4-positive T cell subsets in early breast cancer: a study based on the FinHer trial patient population
Source: Breast Cancer Res. 2018 Feb 26;20:15. doi: 10.1186/s13058-018-0942-x (PMC5827982; doi:10.1186/s13058-018-0942-x)
Supplement: Supplementary file 6 — Table S4. Associations between patient and tumor characteristics and cancer median CD4 expression. (DOCX 16 kb) [file 13058_2018_942_MOESM6_ESM.docx]

**Table S4**. Associations Between Patient And Tumor Characteristics And Cancer Median CD4 Expression

| **Characteristic** | **Cancer CD4 Content**  **≤ Median > Median No. (%) No. (%)** | | ***P*** |
| --- | --- | --- | --- |
|  |  |  |  |
| Tumor size |  |  | 0.056 |
| pT1 | 181 (49.3%) | 186 (50.7%) |  |
| pT2 | 209 (48.4) | 223 (51.6%) |  |
| pT3 | 48 (63.2%) | 28 (36.8%) |  |
| Axillary nodal status |  |  | 0.300 |
| pN0 | 39 (42.4%) | 53 (57.6%) |  |
| pN1 | 385 (50.9%) | 371 (49.1%) |  |
| pN2 | 14 (51.9%) | 13 (48.1%) |  |
| Histological grade |  |  | 0.293 |
| I | 65 (51.2%) | 62 (48.8%) |  |
| II | 183 (52.6%) | 165 (47.4%) |  |
| III | 172 (46.9%) | 195 (53.1%) |  |
| Age at study entry |  |  | 0.090 |
| <50 years | 209 (53.3%) | 183 (46.7%) |  |
| ≥50 years | 230 (47.5%) | 254 (52.5%) |  |
| Estrogen receptor status |  |  | 0.201 |
| Positive | 325 (51.5%) | 306 (48.5%) |  |
| Negative | 114 (46.5%) | 131 (53.5%) |  |
| Progesterone receptor status |  |  | 0.151 |
| Positive | 265 (52.3%) | 242 (47.7%) |  |
| Negative | 174 (47.3%) | 194 (52.7%) |  |
| HER2 status |  |  | 0.197 |
| Positive | 91 (46.0%) | 107 (54.0%) |  |
| Negative | 348 (51.3%) | 330 (48.7%) |  |
| Ki-67 |  |  | 0.390 |
| ≤ 20% (median) | 192 (48.9%) | 201 (51.1%) |  |
| > 20% | 176 (45.7%) | 209 (54.3%) |  |
| Molecular subtype  L |  |  | 0.771 |
| Luminal A-like | 159 (48.3%) | 170 (51.7%) |  |
| Luminal B-like | 76 (51.7%) | 71 (48.3%) |  |
| Triple-negative | 63 (47.7%) | 69 (52.3%) |  |
| HER2-positive | 91 (46.0%) | 107 (54.0%) |  |
| Assigned chemotherapy |  |  | 0.787 |
| Vinorelbine | 214 (49.5%) | 218 (50.5%) |  |
| Docetaxel | 225 (50.7%) | 219 (49.3%) |  |
| Trastuzumab given (if HER2+) cancers) |  |  | ≥ 1.000 |
| Yes | 46 (45.1%) | 56 (54.9%) |  |
| No | 43 (45.7%) | 51 (54.3%) |  |

Abbreviations: CD4, cluster of differentiation 4; HER2, human epidermal growth factor receptor 2.
