# Supplementary material for: Prognostic impact of CD4-positive T cell subsets in early breast cancer: a study based on the FinHer trial patient population
Source: Breast Cancer Res. 2018 Feb 26;20:15. doi: 10.1186/s13058-018-0942-x (PMC5827982; doi:10.1186/s13058-018-0942-x)
Supplement: Supplementary file 7 — Table S5. Univariable Cox regression analysis for distant disease-free survival. (DOCX 16 kb) [file 13058_2018_942_MOESM7_ESM.docx]

**Table S5.** Univariable Cox Regression Analysis for Distant Disease-free Survival

| Tumor/Patient Characteristic | Distant Disease-free Survival  HR (95% CI)  HR (95% CI) | *P* |
| --- | --- | --- |
|  |  |  |
| CXCL13 expression |  |  |
| ≤ Median | Ref. |  |
| > Median | 0.71 (0.51-0.99) | 0.044 |
| FOXP3 expression |  |  |
| ≤ Median | Ref. |  |
| > Median | 1.33 (0.95-1.86) | 0.094 |
| CD4 expression |  |  |
| ≤ Median | Ref. |  |
| > Median | 0.99 (0.71-1.39) | 0.994 |
| Age at study entry |  |  |
| ≥ 50 years | Ref. |  |
| < 50 years | 0.88 (0.65-1.20) | 0.419 |
| Tumor size |  | 0.004 |
| pT1 | Ref. |  |
| pT2 | 1.55 (1.10-2.17) | 0.012 |
| pT3 | 2.22 (1.37-3.67) | 0.002 |
| Axillary nodal status |  | <0.001 |
| pN0 | Ref. |  |
| pN1 | 2.10 (1.07-4.12) | 0.031 |
| pN2 | 5.59 (2.31-13.48) | <0.001 |
| Histological grade |  | <0.001 |
| I | Ref. |  |
| II | 2.67 (1.07-4.12) | 0.006 |
| III | 3.77 (1.90-7.49) | <0.001 |
| ER status |  |  |
| Negative | Ref. |  |
| Positive | 0.56 (0.41-0.77) | <0.001 |
| PR status |  |  |
| Negative | Ref. |  |
| Positive | 0.72 (0.53-0.98) | 0.036 |
| HER2 status |  |  |
| Negative | Ref. |  |
| Positive | 1.74 (1.26-2.41) | 0.001 |
| Ki67 expression |  |  |
| ≤ 20% (median) | Ref. |  |
| > 20% | 1.87 (1.33-2.63) | <0.001 |
| Molecular subtype |  | <0.001 |
| Luminal A-like | Ref. |  |
| Luminal B-like | 2.01 (1.23-3.27) | 0.005 |
| Triple-negative | 3.02 (1.89-4.82) | <0.001 |
| HER2-positive | 2.75 (1.80-4.21) | <0.001 |

Abbreviations: CD4, cluster of differentiation 4; CI, confidence interval; CXCL13, C-X-C motif chemokine ligand 13; ER, estrogen receptor; FOXP3, forkhead box P3; HR, Hazard ratio; HER2, human epidermal growth factor receptor 2; PR, progesterone receptor.
