# Supplementary material for: Prognostic impact of CD4-positive T cell subsets in early breast cancer: a study based on the FinHer trial patient population
Source: Breast Cancer Res. 2018 Feb 26;20:15. doi: 10.1186/s13058-018-0942-x (PMC5827982; doi:10.1186/s13058-018-0942-x)
Supplement: Supplementary file 8 — Table S6. Associations between breast cancer median FOXP3 content and cancer molecular subtype in univariable and multivariable cox regression models for distant disease-free survival. (DOCX 14 kb) [file 13058_2018_942_MOESM8_ESM.docx]

**Table S6.** Associations Between Breast Cancer Median FOXP3 Content And Cancer Molecular Subtype in Univariable And Multivariable Cox Regression Models for Distant Disease-free Survival

| **Molecular subtype** | **Univariable analysis**  **HR (95% CI) *P*** | | **Multivariable analysis***  **HR (95% CI) *P*** | |
| --- | --- | --- | --- | --- |
| Luminal A-like | 0.96 (0.43-2.11) | 0.911 | 0.67 (0.28-1.60) | 0.370 |
| Luminal B-like | 0.78 (0.37-1.66) | 0.524 | 0.75 (0.34-1.64) | 0.473 |
| Triple-negative | 1.37 (0.63-2.96) | 0.426 | 1.54 (0.68-3.47) | 0.300 |
| HER2-positive | 0.93 (0.48-1.81) | 0.831 | 0.85 (0.43-1.69) | 0.647 |

Abbreviations: CI, confidence interval; HER2, human epidermal growth factor receptor 2; HR, hazard ratio.

*Adjusted for patient age at the time of study entry, breast tumor size, axillary nodal status, and histological grade.
