# Supplementary material for: Prognostic impact of CD4-positive T cell subsets in early breast cancer: a study based on the FinHer trial patient population
Source: Breast Cancer Res. 2018 Feb 26;20:15. doi: 10.1186/s13058-018-0942-x (PMC5827982; doi:10.1186/s13058-018-0942-x)
Supplement: Supplementary file 9 — Table S7. Associations between breast cancer median CD4 content and cancer molecular subtype in univariable and multivariable cox regression models for distant disease-free survival. (DOCX 14 kb) [file 13058_2018_942_MOESM9_ESM.docx]

**Table S7.** Associations Between Breast Cancer Median CD4 Content And Cancer Molecular Subtype in Univariable And Multivariable Cox Regression Models for Distant Disease-free Survival

| **Molecular subtype** | **Univariable analysis**  **HR (95% CI) *P*** | | **Multivariable analysis***  **HR (95% CI) *P*** | |
| --- | --- | --- | --- | --- |
| Luminal A-like | 0.79 (0.38-1.68) | 0.541 | 0.87 (0.40-1.87) | 0.717 |
| Luminal B-like | 0.98 (0.46-2.10) | 0.969 | 0.97 (0.45-2.12) | 0.974 |
| Triple-negative | 0.78 (0.39-1.55) | 0.473 | 0.89 (0.43-1.83) | 0.744 |
| HER2-positive | 1.15 (0.63-2.08) | 0.652 | 1.10 (0.60-2.04) | 0.759 |

Abbreviations: CI, confidence interval; HER2, human epidermal growth factor receptor 2; HR, hazard ratio.

*Adjusted for patient age at the time of study entry, breast tumor size, axillary nodal status, and histological grade.
